# The antibacterial effect of aquatic and methanolic extract of *Myrtus communis* on *Actinobacillus actinomycetemcomitans*, *Porphyromonas gingivalis* and *Prevotella intermedia*

**Published:** 2018-08

**Authors:** Leila Rahimvand, Mohammad Niakan, Noushin Jalayer Naderi

**Affiliations:** 1 Faculty of Dentistry, Shahed University, Tehran, Iran; 2 Department of Microbiology, Faculty of Medicine, Shahed University, Tehran, Iran; 3 Department of Oral and Maxillofacial Pathology, Faculty of Dentistry, Shahed University, Tehran, Iran

**Keywords:** Antibacterial activity, *Myrtus communis*, *Actinobacillus actinomycetemcomitans*, *Porphyromonas gingivalis*, *Prevotella intermedia*

## Abstract

**Background and Objectives:**

Anaerobic Gram negative bacteria are the main cause of periodontal destruction. It has been shown that *Myrtus communis* have anti-bacterial activity on Gram positive and Gram negative bacteria. The aim of this study was to determine the antibacterial effect of aquatic and methanolic extract of *Myrtus communis* on some of the oral Gram-negative bacteria.

**Materials and Methods:**

The antibacterial effect of aquatic and methanolic extracts of *Myrtus communis* was determined using disk diffusion method at different concentrations from 10 to 500 mg/ml. The diameter of inhibition zones were determined. The MIC was defined using the standard broth macrodilution method. The results of the study were reported descriptively.

**Results:**

The aquatic extract of *Myrtus communis* from 20 to 500 mg/ml had antibacterial effect on *Actinobacillus actinomycetemcomitans, Porphyromonas gingivalis* and *Prevotella intermedia.* The methanolic extract from 10 to 500 mg/ml had antibacterial effect on *A. actinomycetemcomitans, P. gingivalis* and *P. intermediate*. The MIC was achieved at 10 mg/ml, 10 mg/ml and 10 mg/ml for aquatic and methanolic extracts of *Myrtus communis* on *A. Actinomycetemcomitans, P. Gingivalis* and *P. Intermediate*, respectively.

**Conclusion:**

Aquatic and methanolic extracts of *Myrtus communis* had antibacterial effect on *P. gingivalis, A. actinomycetemcomitans* and *P. intermediate*. Most concentrations of aqueous extract were effective on bacteria, so, providing an alcoholic extract, that is a time consuming and costly method, does not seem necessary.

## INTRODUCTION

The incidence and progression of periodontal disease is causally related to periodontal pathogens. The accumulation of the bacterial plaque starts the periodontal disease. An aerobic bacteria, *Actinobacillus actinomycetemcomitans (Aa), Porphyromonas gingivalis (Pg)* and *Prevotellaintermedia (Pi)* are the main etiologic microbial agents in initiating the periodontitis (1).

Various therapeutic methods have been introduced to eliminate the periodontopathic microflora. Scaling and root planning are the conventional methods in microbial plaque eradication. Using mouthwash is a complementary preventive tool for controlling the oral microflora. Both methods have advantages and disadvantages. Plaque removal by the scaling method needs repeated sessions. Tooth discoloration and unpleasant taste are the most important disadvantages of current mouthwashes (1–2). Using herbal mouthwash is a new idea in therapeutic medicine. The major benefits of herbal irrigates are safety, availability, increased shelf life, cost effectiveness and lack of microbial resistance (3).

*Myrtus* is a genus of one or two species of flowering plants in the *Myrtaceae* family (4). It is a shrub form plant with dark green leaves, large flowers and small bluish-black fruits. A wide range of biologically active compounds such as tannins, flavonoids, coumarins, essential oil, fixed oil, fibers, sugars, citric acid, malic acid and antioxidants are present in the plant. Different parts of Myrtus communis have therapeutic properties. It has been shown that *Myrtus communis* depicts antimicrobial activity on the Gram positive and Gram negative bacteria (5–6). The antibacterial effect of *Myrtus communis* on oral gram negative pathogens has not yet been determined. The aim of this study was to examine the antimicrobial effect of *Myrtus communis* on *Actinobacillus actinomycetemcomitans, Porphyromonas gingivalis* and *Prevotella intermedia*.

## MATERIALS AND METHODS

### Plant Material.

The leaves of *Myrtus communis* was collected from traditional pharmacies (Atari) in Tehran, Iran. Collected sample was verified by the Department of Pharmacognosy, School of Pharmacy, Shahid Beheshti University of Medical Sciences, Tehran, Iran.

### Preparation of extracts: Alcoholic extraction.

500 ml of 70% methanol was added to 100 g of the chopped, powdered *Myrtus communis* in a sterile flask. The mixture was kept for 2 days at room temperature and filtered with No. 1 filter paper with 150 μm diameter (Whatman Co, Germany). The extract was dried in Water bath at 70°C for 1 week. Dried powdered extract was kept at 4°C in a tightly closed vial until used. Different concentrations from 10 to 500 mg\ml were prepared from stock solution in distilled water (7).

### Aqueous extraction.

1,000 ml of sterilized boiling water was added to 100 g of *Myrtus communis*. After 4 hours, the solution was filtered by No. 1 filter paper (Whatman Co, Germany) and then dried in Water bath at 70°C. Different concentrations from 10 to 500 mg\ml were prepared from the stock solution.

### Organisms.

*Actinobacillus actinomycetemcomitans* (ATTC 33384), *Porphyromonas gingivalis* (ATTC 33227) and *Prevotella intermedia* (ATTC 25671) were obtained from the bacterial collection of the Department of Microbiology, Medical Faculty, Shahed University, Tehran, Iran. The bacteria were inoculated into solid and aqueous media containing 41 gr/lit *Brucella* agar, 52 gr/lit BHA, 44 gr/lit anaerobic blood agar, 30 gr/lit Thioglycolate fluid, 29 gr/lit Thio broth and 30 gr/lit Trypticase soy (Merck Co, Germany). Then, the samples were inoculated into 5 mg/ml hemin and yeast extract (Sigma Co, Germany) and kept at anaerobic conditions by Gas pack (Merck Co, Germany) at 37°C. 10 μg/vitamin K and 100 ml/l defibrinated sheep blood were added to anaerobic blood agar (Merck Co, Germany) and kept under anaerobic conditions. Solid and aqueous mediums were kept on plates and tubes, respectively, at 4°C until used.

### Disk diffusion test.

A swab of bacterial suspension was spread on Muller Hinton agar plates (Merck Co, Germany). Sterile blank disks of bacterial suspension (McFarland 0.5 turbidity standard) were prepared. The disks were incubated at 37°C for 72 hours in anaerobic condition. After 24 hrs, the zones of inhibition were measured by using an Antibiotic disk Zone Reader.

### Minimum inhibitory concentration (MIC).

The MIC was determined by the broth dilution method (7). The lowest concentration of extract which had inhibitory effect on organism’s growth was recorded as MIC.

## RESULTS

All concentrations of aquatic extract of *Myrtus communis* from 10 to 500 mg/ml had antibacterial effect on *A. actinomycetemcomitans*. The methanolic extract of *Myrtus communis* in all concentrations from 10 to 500 mg/ml had antibacterial effect on *A. actinomycetemcomitans, P. gingivalis* and *P. intermediate* (Table 1). Table 2 shows the MIC of methanolic and aquatic extracts of *Myrtus communis* on *A. actinomycetemcomitans, P. gingivalis* and *P. intermediate*.

**Table 1.** The inhibition zone diameter of methanolic and aquatic extracts of *Myrtus communis* on *P. gingivalis (Pg)*, *A. actinomycetemcomitans (Aa)* and *P. intermediate (Pi)*

**Concentration**	**Extract**	**10 mg/ml**	**20 mg/ml**	**50 mg/ml**	**100 mg/ml**	**200 mg/ml**	**300 mg/ml**	**400 mg/ml**	**500 mg/ml**
Methanolic	*Pg*	15 mm	16 mm	17 mm	20 mm	20 mm	24 mm	24 mm	26 mm
	*Aa*	12 mm	14 mm	16 mm	18 mm	20 mm	21 mm	22 mm	25 mm
	*Pi*	14 mm	16 mm	20 mm	20 mm	21 mm	23 mm	23 mm	25 mm
Aqueous	*Pg*	0	8 mm	10 mm	16 mm	18 mm	20 mm	23 mm	30 mm
	*Aa*	10 mm	14 mm	15 mm	18 mm	20 mm	21 mm	22 mm	25 mm
	*Pi*	0	12 mm	16 mm	17 mm	17 mm	19 mm	20 mm	22 mm

**Table 2.** Antimicrobial activity of aqueous and methanolic extracts of *Myrtus communis* on *P. gingivalis (Pg), A. actinomycetemcomitans (Aa)* and *P. intermediate (Pi)* by determining MIC

**Concentration**	**Extract**	**10 mg/ml**	**20 mg/ml**	**50 mg/ml**	**100 mg/ml**	**200 mg/ml**	**300 mg/ml**	**400 mg/ml**	**500 mg/ml**
Methanolic	*Pg*	+	-MIC	-MIC	- MIC	- MIC	- MIC	- MIC	- MIC
	*Aa*	+	-MIC	- MIC	- MIC	- MIC	- MIC	- MIC	- MIC
	*Pi*	+	- MIC	- MIC	- MIC	- MIC	- MIC	- MIC	- MIC
Aqueous	*Pg*	+	+	+	- MIC	- MIC	- MIC	- MIC	- MIC
	*Aa*	+	+	- MIC	- MIC	- MIC	- MIC	- MIC	- MIC
	*Pi*	+	- MIC	- MIC	- MIC	- MIC	- MIC	- MIC	- MIC

## DISCUSSION

The study shows that the *Myrtus communis* had antibacterial activity on *A. actinomycetemcomitans, P. gingivalis* and *P. intermediate*. The assessment of antimicrobial activity was based on measurement of inhibition zones formed around the discs. The methanolic extract of *Myrtus communis* produced larger zones of inhibition on *A. actinomycetemcomitans, P. gingivalis* and *P. intermediate.*

*Myrtus communis* has bacteriostatic and bactericidal effect (8–9). The antibacterial effect of *Myrtus communis* on *Salmonella* Typhimurium, *Mycobacterium spp* and *Helicobacter pylori* has been reported (10–12). Fani et al. showed that *Myrtus communis* oil had antibacterial effect on mouth isolated *Streptococcus mutans, Aggregatibacter actinomycetemcomitans, Porphyromonas gingivalis, Candida albicans* and 20 strains of *Streptococcus pyogenes* (13). In another study by Hedayati et al. was shown that the essential oil of *Myrtus communis* had antimicrobial effect on *Porphyromonas gingivalis* (14). The present study showed that the aquatic and methanolic extracts of *Myrtus communis* had antibacterial activity on *A. actinomycetemcomitans, P. gingivalis* and *P. intermediate*. The results of previous studies along with the present study indicate that different forms of *Myrtus communis*, as an oil or extract, have antibacterial effect on oral pathogens and potentially could be an effective ingredient for mouthwashes.

*A. actinomycetemcomitans, P. gingivali* and *P. intermediate* are initiating pathogens of periodontitis. Utilization of mechanical methods and antibiotics for bacterial elimination are not totally effective and have disadvantages. The changing trends in the etio-pathogenesis and prevention of periodontal disease are innumerable. As researches have shown that *Myrtus communis* has antibacterial effect on oral Gram positive (15) and Gram negative pathogens (13), it seems that *Myrtus communis* is a good choice for an herbal-based mouthwash. *Myrtus communis* is an Iranian native herb. It cultivates in Khorasan, Manjil, Kazeron and Sarab regions (16). Easy access to *Myrtus communis* in Iran makes it a profitable choice for use in herbal –based mouthwashes. Application of *Myrtus communis* oil or extract as in local delivery systems like strips, chips, and fibers in treatment of periodontal disease or in combination with regenerative materials to improve periodontal regeneration should be examined. This may help to avoid the side effects of antibiotics in periodontitis. The combined use of *Myrtus communis* and antibiotics could be useful in fighting emerging drug-resistant problem. Since the bacteria were susceptible to all concentrations, it is recommended to use lower concentrations in future studies. Further *in vivo* studies need to be conducted to complete the results about the antibacterial effect of *Myrtus communis*.

In conclusion, the methanolic and aquatic extractions of *Myrtus communis* had antibacterial effect on *A. Actinomycetemcomitans, P. gingivalis* and *P. intermediate*. Most concentrations of aqueous extract of *Myrtus communis* were effective on bacteria, so, providing an alcoholic extract, that is a time consuming and costly method, does not seem necessary.
